# Editorial: Post-pandemic organisational behaviour: mapping the impact of COVID-19 pandemic and Industry 4.0 on employees' behaviour and performance

**DOI:** 10.3389/fpsyg.2023.1251670

**Published:** 2023-08-07

**Authors:** Anastasios Diamantidis, Leslie Thomas Szamosi, Aristea Ilias Ladas, Slobodan Velinov

**Affiliations:** ^1^Department of Production and Management Engineering, Democritus University of Thrace, Xanthi, Greece; ^2^CITY College, University of York Europe Campus, Thessaloniki, Greece; ^3^Grenoble Ecole de Management, Grenoble, Rhône-Alpes, France

**Keywords:** organizational behavior, e-recruitment, telework, organizational culture, leadership, employee performance, Industry 4.0, impact of COVID-19

The COVID-19 pandemic severely disrupted employee behavior and performance worldwide. Adding to this, Industry 4.0 — artificial intelligence, automation, and new technologies — has exasperated these changes to corporate operations. This special issue Research Topic sought out to analyze and shed light on post-pandemic organizational behavior and examine how the COVID-19 pandemic and Industry 4.0 has affected employee behavior and performance.

The pandemic caused by the COVID-19 virus has significantly impacted the methods through which businesses function as well as the organizational behavior of workers. According to research, the move to remote work (teleworking) has had an effect on both the behavior and performance of employees. In this context, the study of Härtel et al. suggests that a person-environment fit viewpoint was found to be the most promising way to widen a focused emphasis on boundary theory-based telework tactics to understand their confusing effects on telework outcomes and adjusting evidence-based best practice telework techniques to teleworkers' specific preferences and requirements (boundary management preferences and telework experience). Moreover, the study of Buzás and Faragó, stressed leadership openness as a key factor to motivate employee voice behavior during work from home situations, a findings that it is in line with the DeRue social interactionist adaptive leadership theory, stating that leadership openness and employee voice mutually are reinforced.

Nevertheless, the pandemic has also had a detrimental effect on the conduct of organizations and the performance of their employees. According to research by Kniffin et al. (2021), employees who are isolated from one another at work and who do not communicate with one another often report lower levels of job satisfaction. Technology, artificial intelligence, and automation are all components of Industry 4.0, and these components have a tremendous influence not only on the behavior of organizations but also on employee performance. Additionally, the study of Doan et al. found that, during the pandemic, there was a decrease in employee satisfaction, motivation and confidence to work, and they highlighted that gender, marital status, and financial stability were found to significantly influence one's inclination to change jobs. According to Brynjolfsson and McAfee (2017), in order for workers to maintain their level of competitiveness in the labor market, they are required to adapt to new technology while simultaneously gaining new knowledge and abilities simply to stay valuable. In this framework the study of Wijaya et al. examined how employer branding and e-recruitment affect millennial ambitions in Indonesia's few e-grocery enterprises; they suggest that E-recruitment platforms failed to forecast millennials' job applications in e-grocery enterprises and that Indonesian companies may need to assess millennials' knowledge with e-recruitment channels, notably corporate websites. They also suggested that employer branding predicted millennials' interest in e-grocery jobs regardless of work experience and concluded that e-recruitment was a branding tool that influenced millennial job searchers and that millennials' intentions were best predicted by the employee value proposition, which prioritized holistic wellbeing.

The pandemic caused by the COVID-19 virus and Industry 4.0 will have a complicated and varied effect on the behavior and performance of employees in the years to come. In order to confront the problems and embrace the chances that have been given, efforts are required from both the management and workforce. In that context the study of Wittmers and Maier on leaders' mental health found that, organizational instrumental support is a moderator for the relation of work intensification and mental illness and, in addition, leaders with a high level of occupational self-efficacy and work intensification had a stronger positive association with exhaustion. Finally, and moreover, the study of Michulek et al. found that organizational culture directly affects leadership, work engagement and work performance, thus stressing the fundamental fact that in times of crisis again organizational culture is the crucial factor, in other words the crucial antecedent for organizational excellence.

## Author contributions

All authors read and approved the final version of the manuscript.
